# New Middle Permian palaeopteran insects from Lodève Basin in southern France (Ephemeroptera, Diaphanopterodea, Megasecoptera)

**DOI:** 10.3897/zookeys.130.1311

**Published:** 2011-09-24

**Authors:** Jakub Prokop, André Nel

**Affiliations:** 1 Charles University in Prague, Faculty of Science, Department of Zoology, Viničná 7, CZ-128 44, Praha 2, Czech Republic; 2 CNRS UMR 7205, Muséum National d’Histoire Naturelle, CP 50, Entomologie, 45 rue Buffon, F-75005 Paris, France

**Keywords:** Insecta, Palaeoptera, Syntonopteridae, Alexrasnitsyniidae fam. n., Parelmoidae, gen. n., sp. n., Middle Permian, palaeodiversity

## Abstract

Three new palaeopteran insects are described from the Middle Permian (Guadalupian) of Salagou Formation in the Lodève Basin (South of France), viz. the diaphanopterodean Alexrasnitsyniidae **fam. n.**, based on *Alexrasnitsynia permiana* **gen. et** **sp. n.**, the Parelmoidae *Permelmoa magnifica* **gen. et** **sp. n.**, and *Lodevohymen lapeyriei* **gen. et** **sp. n.** (in Megasecoptera or Diaphanopterodea, family undetermined). In addition the first record of mayflies attributed to family Syntonopteridae (Ephemeroptera) is reported. These new fossils clearly demonstrate that the present knowledge of the Permian insects remains very incomplete. They also confirm that the Lodève entomofauna was highly diverse providing links to other Permian localities and also rather unique, with several families still not recorded in other contemporaneous outcrops.

## Introduction

Before the tremendous effort of collect of Dr Jean Lapeyrie who brought together a large collection of thousands of fossils, the ‘red’ Middle Permian Salagou Formation (Lodève Basin) was considered devoid of fossils. Palaeoenvironment of Lodève Basin could be characterized as climate controlled playa with ephemeral pools colonized by aquatic clam shrimps (Conchostraca) and tadpole shrimps (Notostraca) together with insects (Lopez et al. 2008). This palaeoenvironment was rich of insects those preservation exhibit considerable high diversity. Representatives of the orders Palaeodictyoptera, Diaphanopterodea, Odonatoptera, Orthoptera, Caloneurodea, Grylloblattodea, Hemiptera, and Glosselytrodea have already been described (see the state of the art in Béthoux 2008; plus Nel pers. comm.).

We describe herein four new fossil insects from the Salagou Formation those correspond to important new taxa. Unfortunately the great majority of this entomofauna is represented by isolated wings. The insect bodies have been destroyed by the abundant necrophagous animals (mainly *Triops* Schrank, 1803) and the decay due to taphonomic process (Gand et al. 1997). This limitation renders difficult the attribution of some fossils, as can be seen below.

## Material and methods

The material described in the present paper comes from the Middle Permian deposits of Salagou Formation found in several localities in the Lodève Basin, Hérault, France (Garric 2000; Béthoux et al. 2002; Nel et al. 2009). Insects are preserved as compressed fossils and deposited in a playa palaeoenvironment. All specimens come from the Lapeyrie collection, currently housed at the Musée of Lodève, France.

The material was observed under stereomicroscope Olympus SZX-9 and venation pattern drawings were drawn directly through stereomicroscope by camera lucida. Photographs were made using digital camera Nikon D80 with macro lens Nikon AF-S VR Micro-Nikkor 105 mm by single sided cross-light exposure.

We follow the wing venation nomenclature of Kukalová-Peck (1991). Abbreviations of wing veins: C costa, ScP subcosta posterior, RA radius anterior, RP radius posterior, MA media anterior, MP media posterior, CuA cubitus anterior, CuP cubitus posterior, AA anal anterior, AP anal posterior.

## Systematic part

### Order Ephemeroptera Hyatt & Arms, 1890. Family Syntonopteridae Handlirsch, 1911

#### Genus indet.

Figs 1A–B

##### Material.

Specimen Ld LAP 483 (Lapeyrie collection, imprint of proximal part of forewing), stored at the Musée of Lodève, France.

##### Age and locality.

Middle Permian, Guadalupian, Lodève Basin, Salagou Formation, Lodève, Hérault, France (Garric 2000; Béthoux et al. 2002).

##### Description.

Counter-imprint of a fore wing without trace of preserved coloration, strongly developed corrugation of longitudinal veins; basal part of fore wing 22.7 mm long and 11.6 mm wide, estimated total length about 45 mm; area between ScP and C rather broad with numerous simple cross-veins; concave ScP straight and basally running close to radial and medial veins; RA nearly straight; RA and RP extremely approximate between wing base and a point situated 17.5 mm distally at which RP strongly diverges from RA; convex MA diverging from MP nearly at right angle and directed towards radial veins, 11.3 mm from wing base; MA distally closely parallel to RA and RP for 5.4 mm, then nearly touching RP at one point; RP emerging 6.4 mm distal of base of MA; concave MP nearly straight; convex CuA diverging from CuP close to wing base and running parallel to medial veins, CuA with two visible terminal branches; simple CuP strongly concave and only weakly curved; anal area partly preserved, first anal vein of neutral polarity ending with two main branches, second anal vein convex and distally pectinate with several branches connected by rather dense network of cells; along posterior wing margin a broad area between CuP and first anal vein; a small pentagonal elongate cell below second anal vein, near wing base.

**Figure 1.**
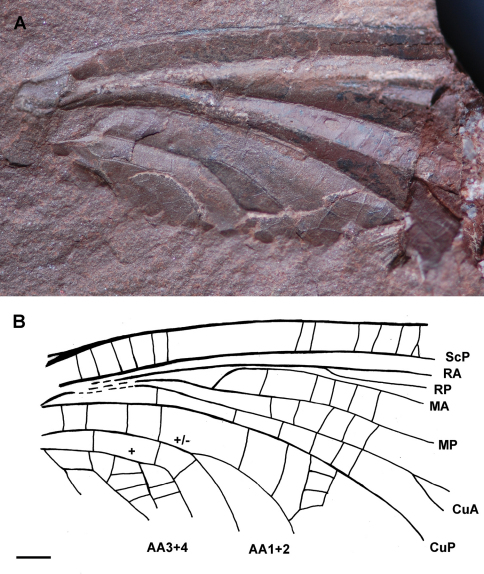
Genus and species indet.(Ephemeroptera: Syntonopteridae), specimen Ld LAP 483: **A** photograph of forewing **B** drawing of forewing (Scale bar represents 2 mm).

##### Discussion.

This fossilbears a combination of the main characters of ephemeropterid family Syntonopteridae, i.e., a strong corrugation of the main longitudinal veins connected by mainly simple transverse crossveins also present in Odonatoptera and some Palaeodictyopterida (e.g., Breyeriidae), MA with a strong anterior curve at its base, shortly connected with RP distally; CuA with a short terminal twigging and anal area with well-defined cell(s). We can argue that this fossil is a forewing fragment for the absence of a characteristic constriction of concave vein IN- between AA1+2 and AA3+4 (Prokop et al. 2010). The specimen can be possibly attributed to a new genus and species for the long part of MA closely parallel to extremely approximate bases of RA and RP; a broad area between CuP and first anal vein near posterior wing margin, and CuP simple (differences with all other genera *Lithoneura* Carpenter, 1938, *Syntonoptera* Handlirsch, 1911, *Anglolithoneura* Prokop & al., 2010, except maybe *Gallolithoneura* Garrouste et al., 2009 based on a rather incomplete wing) (Garrouste et al. 2009). However, we prefer to maintain this specimen as Syntonopteridae gen. et sp. indet. for its incompleteness. Nevertheless this fossil represents the first undisputed record of mayflies from Salagou Formation in the Lodève Basin.

### Order Diaphanopterodea Handlirsch, 1919

#### Family Alexrasnitsyniidae fam. n.

urn:lsid:zoobank.org:act:88B68F64-05B2-4F56-916F-5D7B6E649FB2

http://species-id.net/wiki/Alexrasnitsyniidae

##### Type genus and species.

*Alexrasnitsynia permiana* gen. et sp. n.

##### Diagnosis.

Wing venation only. ScP ending on anterior wing margin near mid part of wing; stems of CuA, M and R very close; CuA, MP and MA diverging at the same point; MA and radial stem very closely parallel; MA distally fused for a short distance with RP; a very broad area between RA and anterior wing margin with several long simple oblique crossveins; a very broad area between CuA and CuP; CuA with weak secondary posterior branches.

#### Genus Alexrasnitsynia gen. n.

urn:lsid:zoobank.org:act:AC781C19-37E1-4F27-B416-E23FEBE9D158

http://species-id.net/wiki/Alexrasnitsynia

##### Type species.

*Alexrasnitsynia permiana* sp. n. by monotypy.

##### Etymology.

 Named after Prof. Alexandr Rasnitsyn.

##### Diagnosis.

 That of the family.

#### Alexrasnitsynia permiana sp. n.

urn:lsid:zoobank.org:act:C0239FF9-E345-4B7A-8146-33A90B85659B

http://species-id.net/wiki/Alexrasnitsynia_permiana

Figs 2A–B

##### Material.

 Holotype LdLAP 318A (Lapeyrie collection, prints of two identical wings), stored at the Musée of Lodève, France.

##### Type strata and locality.

 Middle Permian, Guadalupian, Mérifons Member, Salagou Formation, Lodève, Languedoc, France (Garric 2000; Béthoux et al. 2002).

##### Diagnosis.

That of the family.

##### Description.

Wing 11.8 mm long, 3.9 mm wide; ScP simple, ending in costal margin near mid part of wing, a narrow area between it and C without visible crossveins; area between C and RA relatively broad, 0.6 mm wide, with a row of simple oblique crossveins; RA simple ending on anterior wing margin 0.4 mm from wing apex; RP separating from RA 2.6 mm from wing base; RP with six-seven branches and covering a broad area with series of crossveins; CuA, MP, and MA diverging at the same point, 1.3 mm from wing base; MA very closely parallel to radial stem and reaching RP, fused with it for 0.6 mm, and separating again distally, MA simple, slightly curved; MP simple; CuA with three short distal branches; a series of crossveins between MP and CuA; a broad area between CuA and CuP with crossveins; one anal vein preserved at least.

**Figure 2.**
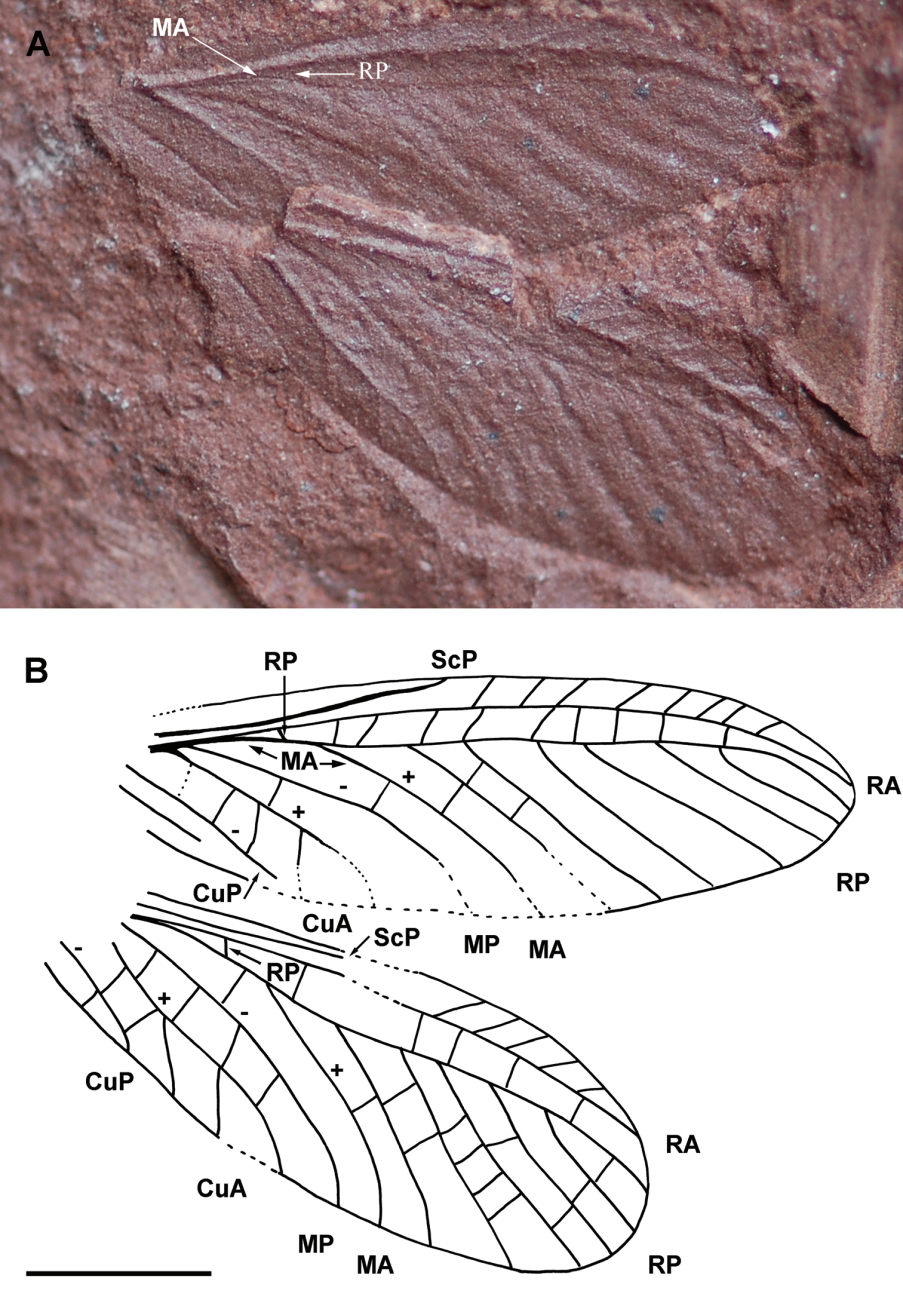
*Alexrasnitsynia permiana* gen. et sp. n. (Diaphanopterodea: Alexrasnitsyniidae fam. n.), holotype LdLAP 318A: A photograph of wings, B drawing of wings (Scale bar represents 3 mm).

##### Discussion.

As the two wings show the same convexity of the veins and are very close, they are likely to be a fore- and a hindwing of the same specimen, but it is impossible to determine which one is the forewing. *Alexrasnitsynia* has a pattern of wing venation characteristic of many Diaphanopterodea (Martynoviidae, Biarmohymenidae, Asthenohymenidae, Rhaphidiopsidae, etc.), i.e. crossveins distinct, archedictyon absent, stems of M and R very close; CuA, MP and MA diverging at the same point; MA very closely parallel with radial stem and distally fused for a short distance with RP. *Alexrasnitsynia* differs from all the known representatives of this order, except *Parelmoa* Carpenter, 1947 and *Permuralia* Sinichenkova & Kukalová-Peck, 1997 in the very broad area between RA and anterior wing margin with several long simple oblique crossveins. It differs from these two last genera in vein MA strongly approximating radial stem, a very broad area between CuA and CuP, and CuA with weak secondary posterior branches (Carpenter 1947, 1992; Kukalová-Peck and Sinichenkova 1992; Sinichenkova and Kukalová-Peck 1997).

Note that the Upper Carboniferous monotypic family Velisopteridae Pinto & Adami-Rodrigues, 1997 is based on a fossil that seems to have none of the diaphanopterid characters listed above (Pinto and Adami-Rodrigues 1997). Its attribution to this order should be verified. The Permian genus and species *Walasua maculata* Tan, 1980 is based on a very fragmentary wing. It has some similarities with *Alexrasnitsynia* in the posterior branches of RP regularly organised, partial fusion of MA with RP, MA closely parallel to R, but structures like area between RA and costal margin, CuA, or CuP are not preserved in *Walasua*, rendering difficult the comparison with *Alexrasnitsynia* (Tan 1980). Nevertheless, the area between RA and RP is broad and with numerous crossveins in *Alexrasnitsynia*, unlike the situation in *Walasua*.

**Figure 3.**
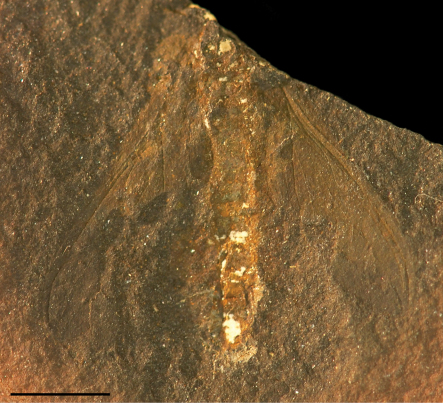
*Sypharoptera pneuma* Handlirsch, 1911(?Diaphanopterodea: Sypharopteridae), holotype YPM No. 0064 (Upper Carboniferous,Carbondale Formation, Mazon Creek (Illinois, USA): photograph (Scale bar represents 5 mm).

*Alexrasnitsynia* bears similar pattern of wing venation to monotypic *Sypharoptera* Handlirsch, 1911, based on *Sypharoptera pneuma* known from the Upper Carboniferous of Mazon Creek (USA). Handlirsch (1911) established a separate order Sypharopteroidea for it, which he thought represented an offshoot of Palaeodictyoptera, with possible relationship to Megasecoptera. Martynov (1938) considered this group an offshoot from Spilapteridae (Palaeodictyoptera) or their ancestors, but did not include it in the latter order. Further placement was done by Rohdendorf (1962) who assigned *Sypharoptera* to Diaphanopterodea on the basis of position of wings. Carpenter first provided revision considering position of *Sypharoptera* in Neoptera contra all previous authors, but resulting with placement to Insecta incertae sedis and later reconsidered as Palaeoptera incertae sedis (Carpenter 1967, 1992). Finally Rasnitsyn (2002: 80) considered *Sypharoptera* to be possibly related to neopterous group Caloneurida on the basis of roof-like wing position, elongate wings with narrow costal space and simple and straight CuA and CuP. However, the holotype of *Sypharoptera* is not in a position that allows determining with accuracy that the wings were in a roof-like position in the living animal (Fig. 3), while the narrow costal space and simple, straight CuA and CuP can be found in many Diaphanopterodea and are not sufficient for an attribution to another clade.

*Sypharoptera* shares with *Alexrasnitsynia* rather short ScP, RA and RP widely separated and connected by a series of transversal crossveins, but the organisation of RP, MA, MP, and CuA at their bases is unknown in the former. So we cannot be sure of its possible affinities with our fossil. In particular, it is not possible to determine on the photograph of the holotype of *Sypharoptera* if it has a MA very closely parallel to radial stem and reaching the short oblique basal part of RP, fused with it for short distance, and separating again distally, as in *Alexrasnitsynia*.

The other structures of wing venation are similar in *Sypharoptera* and *Alexrasnitsynia*, with the main difference in the greater number of branches of RP in the latter (6–7 branches) than in the former (3–4 branches). One might be tempted to consider that *Alexrasnitsynia* and *Sypharoptera* belong to the same family of Diaphanopterodea. Nevertheless several important structures diagnostic to the family to which *Alexrasnitsynia* belongs are unknown in *Sypharoptera*, so we prefer to consider *Alexrasnitsynia* in a separate new family.

#### Family Parelmoidae

Rohdendorf, 1962

##### Type genus.

*Parelmoa* Carpenter, 1947.

##### Other genera.

*Pseudelmoa* Carpenter, 1947, *Permuralia* Sinichenkova & Kukalová-Peck, 1997, *Permelmoa* gen. n.

#### Genus Permelmoa gen. n.

urn:lsid:zoobank.org:act:DAF31992-4002-4C88-BE1D-1ED4B8399DBF

http://species-id.net/wiki/Permelmoa

##### Type species.

*Permelmoa magnifica* sp. n.

##### Etymology.

 Named after the Permian and the genus *Elmoa* Tillyard, 1937.

##### Diagnosis.

 Wing venation only. ScP long, terminating to C close to wing apex; absence of ‘trifurcation between R, M, and CuA near wing base; CuA never touching M; MA and RP never touching.

#### Permelmoa magnifica sp. n.

urn:lsid:zoobank.org:act:6977AE80-1AA2-49C0-92A9-2437B8A41AC7

http://species-id.net/wiki/Permelmoa_magnifica

Figs 4A–B

##### Material.

Holotype Ld LAP 365ab (Lapeyrie collection, imprint and counterimprint of a complete wing), stored at the Musée of Lodève, France.

##### Type strata and locality.

Middle Permian, Lodève Basin, Salagou Formation, Les Vignasses, Lodève, Hérault, France (Garric 2000; Béthoux et al. 2002).

##### Description.

Complete wing without trace of coloration; wing 11.7 mm long and 3.3 mm wide; ScP simple and parallel to RA ending on costal margin close to wing apex (about 0.5 mm); stems R+M basally distinctly curved, with R and M separating about 1.8 mm from wing base; convex simple RA nearly straight, and ending in wing apex; RP separating from RA about 1/3 of wing length, RP with three long branches ending on posterior wing margin; simple convex MA shortly connected to RP by a crossvein; concave MP deeply forked 0.9 mm from division R and M; anterior branch of MP secondary bifurcated, posterior branch simple; simple convex CuA, basally close to R+M; no apparent ‘trichotomy’ R/M/CuA; simple concave CuP; anal area with three or four veins.

**Figure 4.**
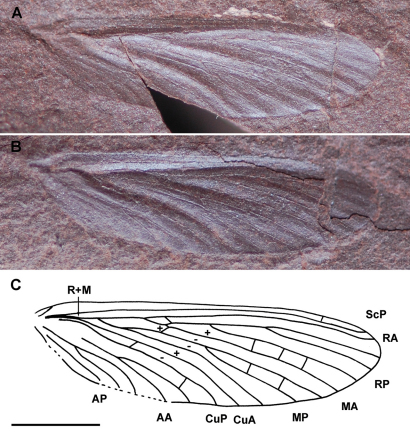
*Permelmoa magnifica* gen. et sp. n. (Diaphanopterodea: Parelmoidae), holotype Ld LAP 365ab: A photograph of wing imprint, B photograph of wing counter-imprint, C drawing of wing (scale bar represents 3 mm).

##### Discussion.

*Permelmoa* is clearly attributable to the Parelmoidae because of combination of the following characters: ScP terminating on C; RP ending with three long branches; MA not coalescent with RP, but shortly connected by crossvein; MP deeply forked. *Permelmoa* differs from *Parelmoa* and *Pseudelmoa* in the absence of trifurcation between R, M, and CuA near wing base, and in the presence of a longer ScP terminating to C close to wing apex. In *Permuralia*, ScP is rather short and terminating on RA, CuA is touching M, and MA and RP are fused for a short distance, unlike in *Permelmoa*.

### Family undetermined

#### Genus Lodevohymen gen. n.

urn:lsid:zoobank.org:act:0D1DBEA0-8B39-4784-907A-5A654B644476

http://species-id.net/wiki/Lodevohymen

##### Type species.

*Lodevohymen lapeyriei* sp. n.

##### Etymology.

Named after Lodève town and Hymen.

##### Diagnosis.

Wing characters only. Presence of basal fusion of M with CuA; broad areas between C and ScP and between ScP and RA without apical ‘pterostigma’-like structure, RP with five posterior parallel branches; strong convexity of basally connected veins R, M, and CuA, forming a curve.

#### Lodevohymen lapeyriei sp. n.

urn:lsid:zoobank.org:act:6FCC725B-B6A5-47D3-854F-04490448BDA7

http://species-id.net/wiki/Lodevohymen_lapeyriei

Figs 5A–B

##### Material.

 Holotype LdLAP 556 (imprint of nearly complete wing, basal part not well preserved, Lapeyrie collection), stored at the Musée of Lodève, France.

##### Type strata and locality.

Middle Permian, Lodève Basin, Salagou Formation, Les Canals, Lodève Basin, Hérault, France (Garric 2000; Béthoux et al. 2002).

##### Description.

 Wing elongated and basally narrow with no trace of coloration preserved, wing fragment about 16.5 mm long, estimated length about 19.5 mm width 4.2 mm in widest part; anterior margin nearly straight, ScP straight ending to C close to wing apex; areas between costal margin and ScP and ScP and RA rather broad; stem of R basally connected to M diverging 1/3 of wing length; division of RA and RP about 8.5 mm from the wing base, RA simple and straight ending in wing apex; RP pectinate with five branches ending on posterior wing margin, crossveins organized in rows between branches of RP; simple MA strongly diverges from MP 1.2 mm from division of stems R and M, and well connected with RP for distance; MP, CuA separates from CuP near wing base, CuA and M basally fused and closely parallel to R, separating 3.0 mm from base of CuA; CuA and CuP simple and straight with a few crossveins between them; anal area reduced, one anal vein present close the wing base.

**Figure 5.**
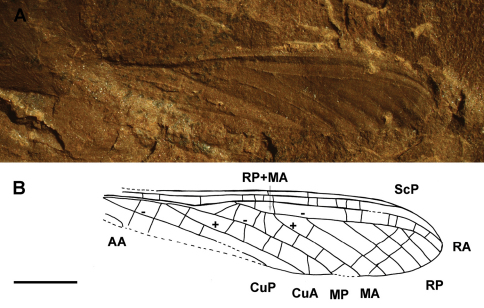
*Lodevohymen lapeyriei* gen. et sp. n.(?Diaphanopterodea), holotype LdLAP 556: A photograph of wing, B drawing of wing (Scale bar represents 3 mm).

##### Discussion.

*Lodevohymen* shares with the diaphanopterodean taxa *Asthenohymen* Tillyard, 1924 (including *Karoohymen* Riek, 1976) (Asthenohymenidae Tillyard, 1924) and *Biarmohymen* Zallesky, 1937 (Biarmohymenidae Zallesky, 1937), but also with the megasecopteran taxa *Permohymen* Tillyard, 1924, *Ivahymen* Martynov, 1932, *Protohymen* Tillyard, 1924 (Protohymenidae Tillyard, 1924), *Scytohymen* Martynov, 1937, *Oceanoptera* Shcherbakov, 2009 (in Shcherbakov et al. 2009), and *Tshekardohymen* Rohdendorf, 1940 (Scytohymenidae Martynov, 1937), the presence of basal fusion of M with CuA (Carpenter 1992; Riek 1976; Shcherbakov et al. 2009). This important character together with a very similar pattern of venation present in all these taxa would suggest that their attribution to different orders is weakly supported.

Nevertheless *Lodevohymen* strongly differs from all these taxa in the broad areas between C and ScP and between ScP and RA without apical ‘pterostigma’-like structure. Other Diaphanopterodea and Megasecoptera have not a long fusion CuA-M in basal part of wing (Carpenter 1992).

The genus *Sunohymen* Hong, 1985, currently included into the Protohymenidae, shares with *Lodevohymen* the rather broad areas between C and ScP and RA, but they differ in RP with only two branches in the former. Also all its structures of basal half of wing are unknown, so that its attribution to the Protohymenidae, family in which M is basally fused to CuA, remains undemonstrated (Hong 1985).

*Lodevohymen* is obviously strongly different from all other Diaphanopterodea and Megasecoptera.

*Lodevohymen* could be better attributed to the Diaphanopterodea rather than to the Megasecoptera for the strong convexity of the basally connected veins R, M, and CuA, forming a curve. Nevertheless, as already noted by Carpenter (1992), it is very difficult to attribute isolated wings of this kind to one of these two orders. It is better to avoid creating a new family as the phylogeny of these two orders is still to be done and the limits of the described families not clearly defined.

## Conclusion

We described in the present study two new representatives of Diaphanopterodea, including a new family, plus one enigmatic taxon that could be either related to this order or to the Megasecoptera, and the first representative of the order Ephemeroptera in the Salagou Formation. These new fossils confirm that this fauna is very rich, diverse and rather unique. It also confirms the general impression that the current knowledge on the Permian insects remains very incomplete. Interestingly, the Coleoptera and other holometabolous orders remain nearly unrecorded in the Lodève basin while these insects are already present in the Lower Permian and dominate all the fossil entomofaunas after the Triassic of Vosges (Papier et al. 2005). This confirms the hypothesis of Nel et al. (2007) about the delayed diversification of the Holometabola after the Permian-Triassic crisis. The Middle Permian appears as a period of renewal of the ‘ancient’, Carboniferous, groups of insects, especially in the palaeopteran clades, before the great extinctions that took place during the period between the Upper Permian and Lower Triassic.

## Supplementary Material

XML Treatment for Genus

XML Treatment for Alexrasnitsyniidae

XML Treatment for Alexrasnitsynia

XML Treatment for Alexrasnitsynia permiana

XML Treatment for Parelmoidae

XML Treatment for Permelmoa

XML Treatment for Permelmoa magnifica

XML Treatment for Lodevohymen

XML Treatment for Lodevohymen lapeyriei
